# Icaritin Preparation from Icariin by a Special *Epimedium* Flavonoid-Glycosidase from *Aspergillus* sp.y848 Strain

**DOI:** 10.4014/jmb.2112.12036

**Published:** 2022-02-11

**Authors:** Zhenghao Wang, Chunying Liu, Hongshan Yu, Bo Wu, Baoyu Huai, Ziyu Zhuang, Changkai Sun, Longquan Xu, Fengxie Jin

**Affiliations:** 1 College of Biotechnology, Dalian Polytechnic University, Qinggong-Yuan No. 1, Ganjingzi-qu, Dalian 116034, P.R. China; 2 School of Life Science and Biotechnology, Liaoning Marine Microbial Engineering and Technology Center, Dalian University, Xuefu-Dajie No. 10, Economic Technological Development Zone, Dalian 116622, P.R. China; 3 Dalian Center for Certification and Food and Drug, Huanghe-Lu No. 888A, Shahekou-qu, Dalian 116021, P.R. China; 4 Research & Educational Center for the Control Engineering of Translational Precision Medicine, Dalian University of Technology, Linggong-ru No. 2, Ganjingzi-qu, Dalian 116024, P.R. China

**Keywords:** *Epimedium* flavonoids-glycosidase, icaritin, epimedin A, B, and C, biotransformation, *Aspergillus* sp.y848

## Abstract

In this study, to obtain icaritin with high pharmacological activities from icariin, which has a content ratio of over 58% in the total flavonoids of *Epimedium* herb, a special *Epimedium* flavonoid-glycosidase was produced, purified and characterized from *Aspergillus* sp.y848 strain. The optimal enzyme production was gained in a medium containing 5% (w/v) wheat bran extract and 0.7% (w/v) *Epimedium* leaf powder as the enzyme inducer, and strain culture at 30°C for 6-7 days. The molecular weight of the enzyme was approximately 73.2 kDa; the optimal pH and temperature were 5.0 and 40°C. The enzyme *Km* and *Vmax* values for icariin were 15.63 mM and 55.56 mM/h. Moreover, the enzyme hydrolyzed the 7-*O*-glucosides of icariin into icariside II, and finally hydrolyzed 3-*O*-rhamnoside of icariside II into icaritin. The enzyme also hydrolyzed 7-*O*-glucosides of epimedin B to sagittatoside B, and then further hydrolyzed terminal 3-*O*-xyloside of sagittatoside B to icarisiede II, before finally hydrolyzing 3-*O*-rhamnoside of icarisiede II into icaritin. The enzyme only hydrolyzed 7-*O*-glucoside of epimedin A or epimedin C into sagittatoside A or sagittatoside C. It is possible to prepare icaritin from the high-content icariin in *Epimedium* herb using this enzyme. When 2.5% icariin was reacted at 40°C for 18-20 h by the low-cost crude enzyme, 5.04 g icaritin with 98% purity was obtained from 10 g icariin. Also, the icaritin molar yield was 92.5%. Our results showed icaritin was successfully produced via cost-effective and relatively simple methods from icariin by crude enzyme. Our results should be very useful for the development of medicines from *Epimedium* herb.

## Introduction

*Epimedium* herb (or *Epimedii* folium, Yinyanghuo in Chinese), the dried leaf of *Epimedium* genus, has been used for more than two thousand years in China, Korea and Japan as a famous traditional medicinal herb used to promote kidney function, build strong bones and muscles, treat impotence and spermatorrhea, and improve the heart [1, 2]. The *Epimedium* genus (recently summarizing) comprises 68 species (57 among these species grow in China), including *Epimedium brevicornum* Maxim, *E. alpinum* L, *E. Pubigerum* (DC.) Morren & Decaisne, *E. pinnaturn* Fisch, *E. perralderianum* Cosson, *E. diphyllum* Lodd, *E. grandiflorum* Morr, *E. macrosepalum* Stearn, *E. elatum* Morren & Decaisne, *et al*. However, only four types of dried leaf, from *E. brevicornum* Maxim, *E. pubescens* Maxim, *E. sagittatum* Maxim, and *E. koreanum* Nakai, are included in the Chinese Pharmacopoeia [3].

**Fig. 1:**
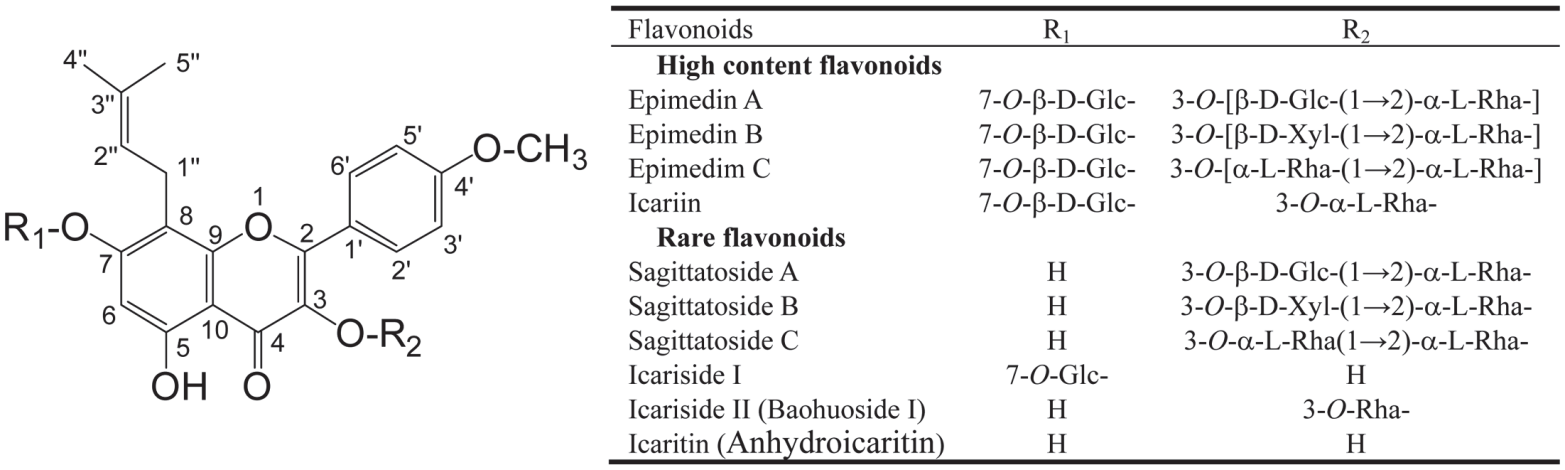
Structures of main *Epimedium* flavonoids. Glc, glucoside; Rha, rhamoside; Xyl, xyloside.

The major active components of *Epimedium* herb are flavonoids with more than 70 types [1, 3]. The total flavonoid content of *Epimedium* herb is over 5% (w/w), but more than 80%-85% (w/w) of these flavonoids are four kinds of 8-isopentene-flavonoids include epimedin A, epimedin B, epimedin C and icariin, while the flavonoid content radio is 58.5% for icariin, 24.5% for epimedin C, 11% epimedin B, and 5.92% epimedin A. The remaining flavonoids are lower in content [4, 5]. The structures of the main *Epimedium* flavonoids are shown in Fig. 1. Epimedin C, B, and A have three glycosides, glucoside, rhamnoside and xyloside, while icariin has two glycosides, rhamnoside and glucoside, and icaritin (anhydroicaritin) is an aglycone without glycoside.

*Epimedium* flavonoids and these aglycones (icaritin or anhydroicaritin) exhibit very useful pharmacological properties that exert immunomodulatory, anti-inflammatory, anti-aging, and anti-tumor activities while also inhibiting osteoporosis, Alzheimer's disease and cerebral ischemia [6]. Modern pharmacological studies suggest that the sugar moieties of *Epimedium* flavonoids are closely associated with their physiological activity; the pharmacological activity of a *Epimedium* flavonoid increases as the number of glycosides decreases, while the pharmacological activity of icaritin is better than that of icariin and other *Epimedium* flavonoids [7, 8]. When taken orally, icariin and *Epimedium* flavonoids with two or three glycosides cannot be directly absorbed by the human body. The glycosides of icariin and *Epimedium* flavonoids are hydrolyzed by intestinal bacteria and digestive enzymes in the human gastrointestinal tract into icaritin, which is easily absorbed, and the icaritin exhibits very useful pharmacological activities. But, the efficiency of this biotransformation of *Epimedium* flavonoids is poor, and the icariin bioavailability of oral absorption is only 12%. Its effect is mainly through metabolism into icaritin, which exhibits a prominent role [9-12]. Icaritin (anhydroicaritin), whose structure is easily absorbed, exerts a variety of very useful pharmacological activities [12, 13] for the treatment of various cancers [14] and osteoporosis [15], promotion of nerve cell differentiation and inhibition of Alzheimer’s disease [8, 16], inflammation, impotence, and angiocardiopathy [12, 13]. However, *Epimedium* herb does not contain much icaritin. Therefore, the transformation of major *Epimedium* flavonoids with low activity and poor absorption into highly active and easily absorbed, rare flavonoids such as icaritin is very useful for the development of *Epimedium* herb.

To obtain icaritin, icariin was hydrolyzed by inorganic acid, but this method produces a lot of contamination and many by-products [17]. The chemical synthesis of icaritin from anhydrous phloroglucin was reported [18], but the icaritin yield was only 4.2% and this process also caused contamination. Bioconversion studies of *Epimedium* flavonoids into icaritin and rare *Epimedium* flavonoids using enzymes have attracted wide attention. For instance, an icariin-glycosidase from *Absidia* sp.E9r strain was purified and the enzyme molecular weight was about 65 kDa [19]. Gao *et al*. analyzed the conversions of epimedin C, B, A and icariin by crude snailase, which gradually hydrolyzed the 7-*O*-β-glucoside of epimedin C, epimedin B, epimedin A or icariin into icariside II, sagittatoside C, sagittatoside B, or sagittatoside A, respectively. The produced intermediates further hydrolyzed into icaritin [20]. Fu *et al*. biotransformed a mixture of epimedin C, B and A into icariin by the enzyme from Aspegillus sp.y39 strain for use in increasing the icariin extraction from *Epimedium* herb [21], but their study did not involve icaritin preparation. Li *et al*. reacted icariin to icaritin using a homemade β-glycosidase, but the enzyme was not characterized [22]. Xie *et al*. hydrolyzed epimedin C into icaritin using two recombinant enzymes of hyperthermophilic α-L-rhamnosidase and thermostable β-glucosidase [23], but the substrate was only epimedin C, and they did not use any high-content icariin, epimedin B or epimedin A from *Epimedium* herb. Zhang *et al*. bioconverted the total flavonoid extract (containing icariin, and epimedin C, B and A) of *Epimedium* into icaritin with two thermostable glycosidases from *Dictyoglomus thermophilum* DSM3960, but the icaritin weight-yield low at 4%, *i.e.*, only 0.20 g/l icaritin was obtained from 5 g/l of the total flavonoid extract of *Epimedium* [24].

Here, to obtain icaritin from icariin, which has a content ratio of over 58% in the total flavonoids of *Epimedium* herb, by enzyme, a special *Epimedium* flavonoid-glycosidase was produced, purified and characterized from *Aspergillus* sp.y848 strain. Then, using the same strain, we were able to prepare icaritin from icariin at low cost.

## Materials and Methods

### Materials

The *Aspergillus* sp.y848 strain was previously isolated from Chinese koji (daqu in Chinese) [25-28]. Standards such as icaritin, icariin, epimedin C, epimedin B and epimedin A, sagittatoside C, sagittatoside B, sagittatoside A, icariside I and icariside II were obtained from Nanjing Guangrun Biotech Co., Ltd. (China) and Chemgdu Purifa Tech Co., Ltd. (China). The substrate icariin was obtained from Shanxi Kangsheng Biotech Co., Ltd. (Xi 'an, Shanxi Province, P.R. China). A 60-F_254_ silica gel plate (Merck, Germany) was used for TLC (thin-layer chromatography) analysis. The marker proteins including lysozyme (14.3 kDa), trypsinogen (24 kDa), glyceraldehyde-3-phosphate (36 kDa), glutamic dehydrogenase (53 kDa), albumin (66 kDa) and phosphorylase (97 kDa) are products from Takara Bio Inc., (Japan).

### Enzyme Production and Purification

To obtain the enzyme for biotransformation of *Epimedium* flavonoids into icaritin, the *Aspergillus* sp.y848 strain was cultured using the reference method of the *A. niger* b.48 [27] and *Aspergillus* sp. agl-84 strains [28] with leaf powder of *Epimedium* herb as an enzyme inducer to get the crude enzyme solution. Then, the crude enzyme was fractioned and purified with a column of DEAE-cellulose DE-52 (Whatman, USA) according to references [27, 28]; the fractions were 3 ml/tube. Enzyme activities of each fraction were examined using icariin as a substrate, respectively. The enzyme protein purity of fractions was also analyzed: the separated fractions were freeze-dried and dissolved with distilled water (1/5 to 1/10 of original volume), then the enzyme protein purity was examined using PAGE, SDS-PAGE [29, 30] and protein HPLC, respectively.

To be safe, the *Epimedium* flavonoid-glycosidase was further purified. The enzyme band of PAGE gel was cleaved, dissolved in 0.02 M (pH 5.0) acetate buffer, and then non-dissolved material was removed using the freeze-centrifugation method to get more pure *Epimedium* flavonoids-glycosidase. The purity of more purified enzyme was again examined by SDS-PAGE and protein HPLC.

The protein concentration of *Epimedium* flavonoid-glycosidase was determined using the Folin phenol reagent. The protein standard was the bovine serum albumin [26-28].

The enzyme molecular weight was measured by SDS-PAGE [30]. The marker proteins were trypsinogen (24 kDa), glyceraldehyde-3-phosphate (36 kDa), glutamic dehydrogenase (53 kDa), albumin (66 kDa) and phosphorylase (97 kDa). The protein bands were visualized using Coomassie brilliant blue R-250. The enzyme molecular weight was calculated according to the mobility of marker proteins based on plotting the log of the marker protein molecular weight [30].

### Epimedium Flavonoid-Glycosidase Analysis and Properties

The effects of temperature and pH on purified *Epimedium* flavonoid-glycosidase from *Aspergillus* sp.y848 strain reaction were analyzed. The reaction pH was fixed to 4.0, 4.5, 5.0, 5.5, 6.0 and 7.0; the reaction temperature was fixed to 35, 40, 45, 50, 55 and 60°C. Under these conditions, the enzyme was reacted with 0.25% (w/v) icariin for 12 h.

The activity of *Epimedium* flavonoid-glycosidase from *Aspergillus* sp.y848 strain was determined using 0.5%(w/v) icariin in 0.02 M (pH 5.0) acetate buffer. Then, 0.1 ml of enzyme solution was mixed with 0.1 ml of different substrate solution (final 0.25% substrate), and reacted at 40°C for 1.0 to 6 h, respectively. After that, 0.2 ml of water-saturated *n*-butanol was added to the reaction solutions to stop the reaction, and the reaction products and substrate in the *n*-butanol layer were determined with the TLC method. TLC spots of substrate and products were scanned by a Shimadzu CS-930 spectrophotometer (Shimadzu Corp., Japan) to calculate the conversions of icariin [26-28].

One unit of *Epimedium* flavonoid-glycosidase was defined as the amount of enzyme that reduces 1 nmol of icariin per minute.

The Michaelis maximal reaction velocity (*V_max_*) and Michaelis constant (*K_m_*) for *Epimedium* flavonoid-glycosidase from *Aspergillus* sp.y848 strain were measured using fully mixed icariin at the concentrations of 12.5, 15.4, 20.0, 28.6, and 50.0 mM in 0.02 M (pH 5.0) acetate buffer. The enzymatic reaction was performed at 40°C and pH 5.0 for 0.5, 1.0, 1.5, 2.0 and 3.0 h, respectively. The icariin conversion velocities were obtained according to the TLC spot ratio of substrates and products in enzyme reactions, respectively. The *V_max_* and *K_m_* were calculated by the Linweaver–Burk plots [31].

### Purified Enzyme Hydrolysis on the Glycosides of Icariin, Epimedin C, B and A

The hydrolysis of *Epimedium* flavonoid-glycosidase from *Aspergillus* sp.y848 strain on the glycosides of icariin, epimedin C, B and A was determined using 0.5% (w/v) icariin, and 0.4% epimedin C, epimedin B or epimedin A in 0.02 M acetate buffer (pH 5.0), respectively. Then, 0.1 ml enzyme solution was mixed with 0.1 ml of different substrate solution (final 0.25%, 0.2% substrate), and reacted at 40°C for 3 to 6 h, respectively. Following that, 0.2 ml of water-saturated *n*-butanol was added to the reaction solutions to stop the reaction, and the reaction products and substrate in the *n*-butanol layer were determined by theTLC [26-28] and HPLC methods, respectively.

### Icaritin Preparation from Icariin by Crude Enzyme

The optimal temperature, pH, substrate concentration, and reaction time of the crude enzyme reaction were determined. The temperature was fixed to 35, 40, 45, 50, 55, and 60°C. The pH was fixed to 4.0, 4.5, 5.0, 5.5, 6.0, and 7.0. The concentration of substrates including icariin was fixed to 1, 2, 3, 4, 5, and 6% (w/v), and then each sample was mixed with same volume of crude enzyme [final substrate concentration: 0.5, 1.0, 1.5, 2.0, 2.5, and 3.0% (w/v)] and reacted at 40°C for 20 h, respectively. In the examination of enzyme reaction time, the reaction time was fixed at 3, 5, 7, 9, 11, 13, 15, 18, 21, and 24 h at 40°C, respectively.

Icaritin was prepared from icariin at the optimal reaction conditions as given above. Then, 10 g icariin was dissolved in 200 ml of 0.02 M acetate buffer (pH 5.0), and mixed with 200 ml crude enzyme. The mixtures were reacted at 40°C for 18-20 h by 60-70 rpm stirring. After reaction, the precipitated icaritin was collected by centrifugal freeze-drying and then washed three times with water before drying to get crude icaritin. The crude icaritin was dissolved in 10× tetrahydrofuran (w/v) and filtered to remove un-dissolved material. Then, 50%methanol was gradually added to icaritin tetrahydrofuran solution to precipitate icaritin. The precipitated icaritin was collected by freeze-centrifugation, washed three times with cold 50% (v/v) methanol, and dried to get pure icaritin. The purity of the icaritin product was determined by HPLC. The experiment was repeated three times.

### TLC and HPLC Analysis of Substrate and Products, and Product NMR Analysis

The 60-F_254_ silica gel plate was used in the TLC analysis of *Epimedium*-flavonoid substrates and products. The developing solvent was ethyl acetate: butanone: methanol: water = 8: 7: 1: 1 (v/v/v/v), and the color of the plate spots was rendered at 280 nm ultraviolet. The TLC spot ratios of substrate and products were measured by scanning with a Shimadzu CS-930 TLC Scanner (Shimadzu, Japan) [26-28].

HPLC analysis of *Epimedium*-flavonoid substrates and products in enzyme reaction was tested by a Waters 2695 Separations Module (Waters, USA) with a Waters 2996 Photodiode Array Detector. The column was a Zhonghuida C18 (5 μm, φ 4.6 mm × 250 mm); the detection wavelength was 273 nm. The mobile phase for substrates and products of the enzyme reaction was A (acetonitrile) and B (water): 0-8 min, A 17% to 27% (v/v); 8-32 min, A was 27% (v/v); 32-60 min, A 27% to 85% (v/v); 60-70 min, A 70% to 80% (v/v); and 70-80 min, A 85% to 100% (v/v). The flow rate was 1 ml/min and the injected volume was 10 μl. The column temperature was 35°C. However, the retention time of the icaritin product was long in the HPLC mobile phase, in which methanol: tetrahydrofuran: 0.1% phosphoric acid water = 35: 26: 39 was used to determine the enzymatic icaritin product. The flow rate was also 1 ml/min and the injected volume was 10 μl.

HPLC enzyme protein purity was determined by chromatographic column (TOSOH TSK-Gel-2000 SW) (φ7.8 mm ×300 mm) from TOSOH ASA Corp., Japan. The acquisition wavelength was 280 nm. The mobile phase was 0.02 M (pH 6.7) phosphate buffer containing 0.05% (w/v) sodium azide. The flow rate of mobile phase was 1.0 ml/min, and the injected volume was 100 μl [27, 28].

The structure of the icaritin product was measured using the NMR method, whereby the icaritin was dissolved in Pyridine-*d*_5_, and the spectra of NMR were analyzed by a Bruker Avance 600 NMR Spectrometer (^13^C, 150 MHz; ^1^H, 600 MHz) (Switzerland).

## Results

### Enzyme Production and Purification

*Epimedium* flavonoid-glycosidase was produced by the culture of *Aspergillus* sp.y848 strain. The strain was cultured in a medium containing 0.2, 0.4, 0.5, 0.6, 0.7, 0.8, or 1% (w/v) *Epimedium* leaf powder as the enzyme inducer; and 4, 5, and 6% (w/v) of wheat bran extract at 28-30°C for 5-8 days by 60-70 rpm stirring, respectively. Optimal enzyme production was gained in medium containing 5% (w/v) wheat bran extract and 0.7% (w/v) *Epimedium* leaf powder, with the strain culture at 30°C for 6-7 days (Fig. S1). This culture of *Epimedium* flavonoid-glycosidase production was similar to that of other flavonoid-glycosidases such as baicalin-β-D-glucuronidase [27].

After the strain was cultured, the cells of the culture were removed by freeze-centrifugation. Then, a 3× volume methanol was added to the supernatant to precipitate enzyme protein, which was collected with the freeze-centrifugation and dissolved in 1/10 enzyme original culture volume of 0.02 M (pH 5.0) acetate buffer. The un-dissolved materials of the solution were removed by freeze-centrifugation to obtain crude enzyme solution. Thus, the special *Epimedium* flavonoid-glycosidase, which could hydrolyze the clycosides of icariin, was produced via the culture of *Aspergillus* sp.y848 strain at 30°C for 6-7 days in a medium containing 5% (w/v) wheat bran extract and 0.7% (w/w) *Epimedium* leaf powder (enzyme inducer).

**Fig. 2:**
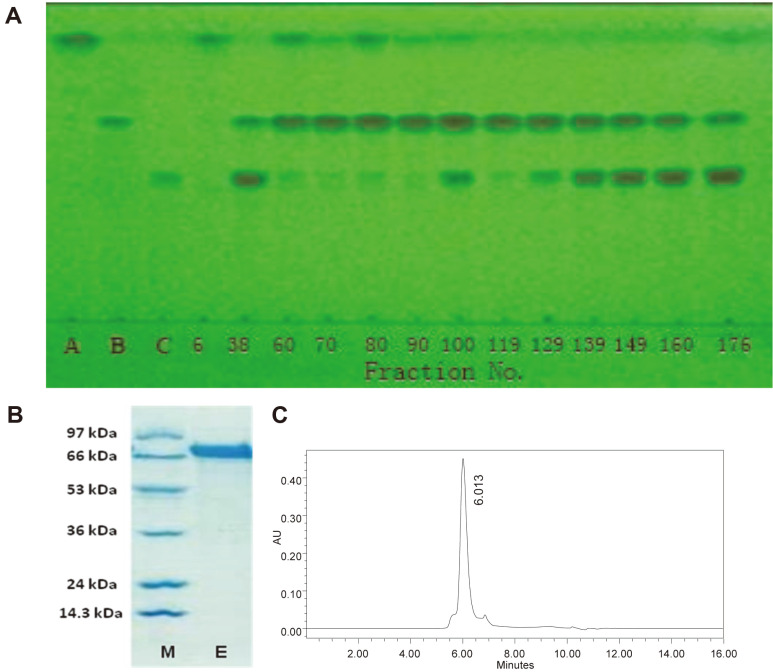
Incariin hydrolysis of enzyme fractions from DEAE-cellulose column in TLC, and purified enzyme in SDS-PAGE and HPLC. (**A**) Icariin hydrolysis of the enzyme fractions 6 to 176 from DEAE-cellulose column in TLC. 0.25% icariin was reacted at 40°C and pH 5.0 for 12 h. A, icariin; B, icariside II, l C, icaritin; 6 to 176, fraction number. Developing solvent, ethyl acetate: butanone: methanol: water = 8: 7: 1: 1 (v/v/v/v); rendering color in 280 nm ultraviolet. (**B**) Purified enzyme of 74 to 84 fractions in SDS-PAGE; E, purified enzyme; M, marker proteins including phosphatase b (97 kDa), bovine serum albumin (66 kDa), glutamic dehydrogenase (53 kDa), glyceralde-3-phosphate (36 kDa), trypsin inhibitor (24 kDa) and lysozyme (14.3 kDa). (**C**) Purified enzyme of 74 to 84 fractions in protein HPLC (73.2 kDa).

In the purification of the special *Epimedium* flavonoid-glycosidase from *Aspergillus* sp.y848 strain, 10 ml of crude enzyme was eluted on a column (φ2.0 × 11.0 cm) of DEAE-Cellulose DE-52 to adsorb enzyme protein. Then, the column was washed by 30 to 50 ml of 0.02 M (pH 5.0) acetic buffer to remove non-adsorbed protein. The enzyme protein of the column was stepwise fractionated and eluted with 100 ml each of 0.06, 0.12, 0.18, 0.24, 0.30, 0.36, 0.42, and 0.50 M KCl in 0.02 M acetate buffer (pH 5.0) and each fraction amounted to 3.0 ml/tube. The enzyme activities of each fraction were analyzed using 0.25% (w/v) icariin solution in 0.02 M (pH 5) acetic buffer, respectively. Following this, 0.1 ml fractions were mixed with 0.1 ml 0.5% (w/v) of icariin and reacted at 40°C for 4-12 h and the icariin hydrolysis of fractions was determined by TLC (Fig. 2A) while the protein purity of fractions was determined by SDS-PAGE (Fig. S2) respectively. The experiment results showed that the enzyme of the 6, 60, 70, 80, 90, and 119 fractions completely hydrolyzed icariin into intermediate product-icariside II and final product-icaritin; but the others did not completely hydrolyze icariin (Fig. 2A). Among the SDS-PAGE of fractions (Fig. S2), fraction 6 was protein mixture, which was similar to crude enzyme, while fraction 119 was also protein mixture. Fractions 60 and 90 contained a small amount of other proteins, and only fraction 80 showed a single band in SDS-PAGE (Fig. S2). Moreover, the protein of fractions 76 to 84 showed almost a single band in the SDS-PAGE (Fig. 2B) and PAGE, and a single peak in protein HPLC (Fig. 2C) to demonstrate that the isolated protein of these fractions was a pure enzyme.

In careful order, the enzyme was further purified. The enzyme band of the PAGE gel was cleaved, and dissolved in 0.02 M acetate buffer (pH 5.0). After removing the non-dissolved material with centrifugation, the further purified enzyme protein was also one band in SDS-PAGE and one peak in protein HPLC; the specific enzyme activity of further purified enzyme was almost the same as that of the enzyme purified by DEAE-Cellulose DE-52 column.

Thus, the *Epimedium* flavonoid-glycosidase was almost purified by DEAE-cellulose DE-52 column alone. At this step, the 1701 U pure enzyme was obtained from 100 ml of 31500 U crude enzyme. Pure enzyme yield was only 5.3% (U/U) and the enzyme specific activity was increased from 439 U/mg to 8237 U/mg by 14.3-fold (The Table was omitted).

The enzyme molecular weight was calculated according to the mobility of marker proteins based on plotting the log of the marker protein molecular weight [30] including lysozyme (14.3 kDa), trypsin inhibitor (24 kDa), glyceraldehyde-3-phosphate (36 kDa), glutamate dehydrogenase (53 kDa), bovine serum albumin (66 kDa) and phosphatase b (97 kDa). Purified enzyme protein of 74 to 84 fractions was one band in SDS-PAGE and PAGE, and a single peak in protein HPLC (Fig. 2C). The molecular weight of the purified *Epimedium* flavonoid-glycosidase from *Aspergillus* sp.y848 strain was approximately 73.2 kDa (Fig. S3), which differed from that of icariin-glycosidase from *Absidia* sp.E9r strain at 65 kDa [19], and also differed from that of baicalin-β-D-glucuronidase from *Aspergillus niger* b.48 strain at 45 kDa [27]. The purification experiment of *Epimedium* flavonoid-glycosidase from *Aspergillus* sp.y848 strain was repeated 5 times, and the results were basically identical.

### Purified Enzyme Some Properties

**Fig. 3:**
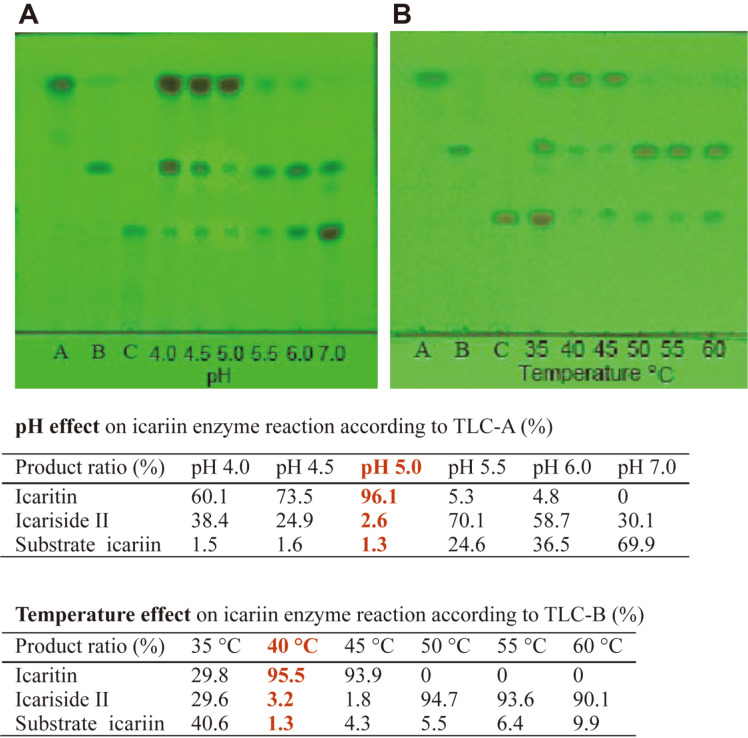
Effects of pH and temperature on glycoside hydrolysis on icariin by purified enzyme. (**A**) pH effect, 0.25% icariin was reacted in different pH by enzyme at 40°C for 12 h. (**B**) Temperature effect. 0.25% icariin was reacted in different temperature by enzyme at pH 5.0 for 12 h. A, icaritin; B, icariside II; C, icariin. Developing solvent, ethyl acetate: butanone: methanol: water = 8: 7: 1: 1 (v/v/v/v); rendering color in 280 nm ultraviolet.

The optimal pH and temperature of the glycoside hydrolysis on icariin by purified enzyme were determined. The pH was fixed to 4.0, 4.5, 5.0, 5.5, 6.0, and 7.0; the temperature was fixed to 35, 40, 45, 50, 55, and 60°C. Under these conditions, 0.25% (w/v) icariin was reacted for 12 h. The optimal pH for icariin was 5.0 (Fig. 3A) and the optimal temperature was 40°C (Fig. 3B). The purified enzyme was very stable, but when the enzyme was incubated at pH 5.0 and 40-45°C for 48 h, the enzyme activity barely decreased.

The effect of metallic ions on the activity of purified enzyme was then examined. Ca^2+^, Mg^2+^, K^+^ and Na^+^ ions have hardly any on enzyme activity. Fe^3+^ ion and more than 10 mM/l of Cu^2+^ ion have a negative effect. More than 50 mM/l of Zn^2+^ ion inhibits enzyme activity. (Table S1).

To determine the kinetic parameters of *Epimedium* flavonoid-glycosidase, the *Vmax* (maximal reaction velocity) and *Km* (Michaelis constant) were examined with mixed icariin at concentrations of 12.5, 15.4, 20.0, 28.6, and 50.0 mM in 0.02 M (pH 5.0) acetate buffer. The solutions was reacted at 40°C for 0.5, 1.0, 1.5, 2.0, and 3.0 h, respectively. The conversion velocities of icariin were calculated based on the TLC spot ratio of substrate flavonoids and the product flavonoids in different enzyme reactions, respectively (Fig. S4). According to Lineweaver–Burk plot [31] of 1/*V* against 1/[*S*], the *V_max_* and *K_m_* for icariin were 55.56 mM/h and 15.63 mM; based on the Michaelis–Menten equation, the enzyme reaction velocities (*V_o_*) in 5 mM substrate were 13.5 mM/h for icariin (Fig. S4).

Therefore, the optimal pH of the purified *Epimedium* flavonoid-glycosidase from *Aspergillus* sp.y848 strain was 5.0 for icariin, the optimal temperature was 40°C; and the *V_max_* and *K_m_* for icariin were 55.56 mM/h and 15.63 mM.

### Purified Enzyme Hydrolysis on the Glycosides of Icariin, Epimedin C, B and A

**Fig. 4:**
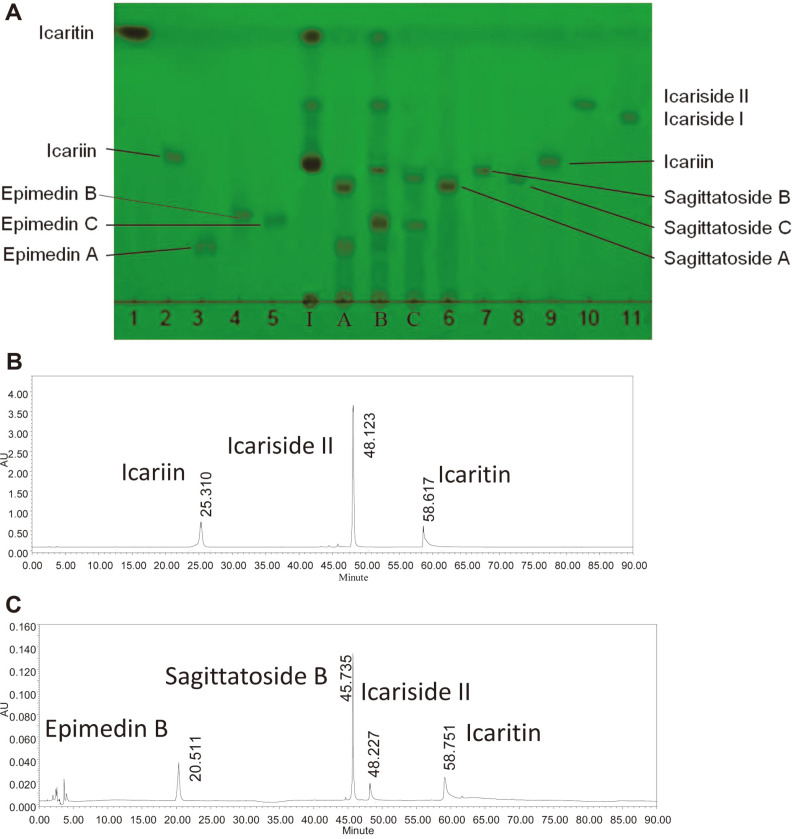
Hydrolysis on the glycosides of icariin, epimedin A, epimedin B and epimedin C in TLC and HPLC by purified *Epimedium* flavonoid-glycosidase from Aspegillus sp.y848 strain. (**A**) Hydrolysis on glycosides of icariin, epimedin A, epimedin B and epimedin C in TLC by purified enzyme. 1 to11, standard iacariin, icaritin, epimedin A, epimedin B and epimedin C, sagittatoside A, sagittatoside B, sagittatoside C, icariin, icariside I and II, icaritin. I, products from 0.25% icariin reacting at 40°C and pH 5.0 for 4 h by enzyme. A, product from 0.2% epimedin A reacting at 40°C and pH 5.0 for 3 h; B, product from 0.2% epimedin B reacting for 6 h; C, product from 0.2% epimedin C reacting for 3 h. Developing solvent, ethyl acetate: butanone: methanol: water = 8: 7: 1: 1 (v/v/v/v); rendering color in 280 nm ultraviolet. (**B**) Hydrolysis on glycosides of icariin in HPLC by purified enzyme. 0.25% icariin was reacted at 40°C and pH 5.0 for 6 h by purified enzyme. (**C**), Hydrolysis on glycosides of 0.2% epimedin B at 40°C and pH 5.0 for 6 h by purified enzyme in HPLC.

The enzyme optimal pH and temperature for the substrates of epimedin C or B or A were also 5.0 and 40°C, respectively. The hydrolysis of the purified *Epimedium* flavonoid-glycosidase from *Aspergillus* sp.y848 strain (above fractions from 76 to 84) on the different glycosides of icariin, epimedin C, B, and A were examined, respectively. First, 0.1 ml of 0.5% (w/v) of icariin, and 0.4% epimedin A, epimedin B and epimedin C were mixed with 0.1 ml purified enzyme (final 0.25% and 0.2% substrate concentration) and reacted at 40°C for 1, 3, 6, and 12 h, respectively. Then, 0.2 ml of water-saturated *n*-butanol was added, and reaction products in the *n*-butanol layer were determined using the methods of TLC and HPLC by comparison with the standard *Epimedium* flavonoids, respectively, as shown in Fig. 4.

**Fig. 5:**
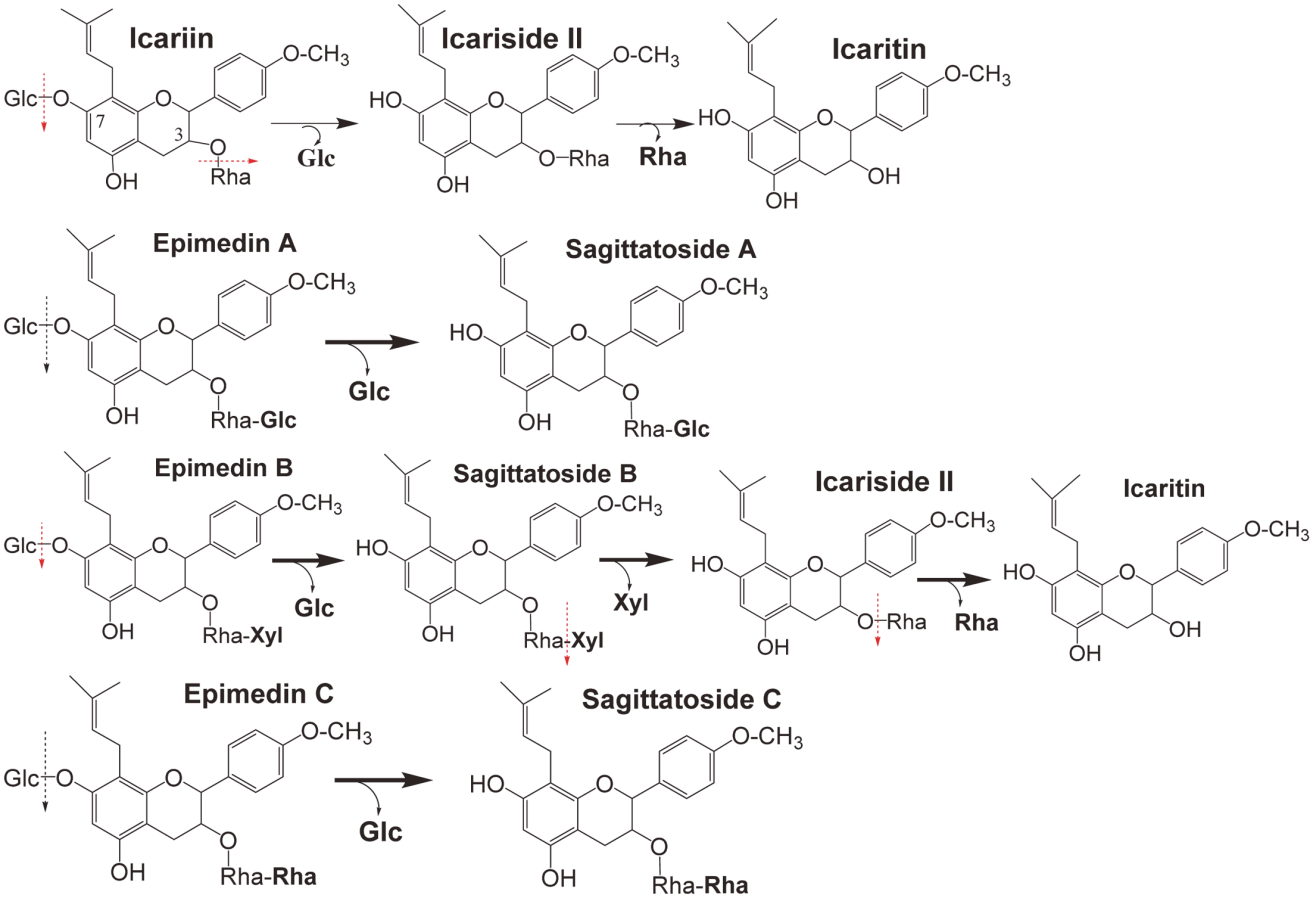
Hydrolysis pathways on the multi-glycosides of icariin, epimedin A, B and C by special *Epimedium* flavonoid-glycosidase from *Aspergillus* sp.y848 strain. Glc, glucoside; Xyl, xyloside; Rha, Rhamnoside.

As shown in Fig. 4, the purified *Epimedium* flavonoid-glycosidase from *Aspergillus* sp.y848 strain first hydrolyzed the 7-*O*-β-D-glucosides of icariin into icariside II, and finally hydrolyzed 3-*O*-α-L-rhamnoside of icariside II into icaritin (Fig. 4A, column I; Fig. 4B). The purified enzyme also hydrolyzed 7-*O*-β-D-glucosides of epimedin B to sagittatoside B, then hydrolyzed terminal 3-*O*-β-D-xyloside of sagittatoside B to icarisiede II, and finally hydrolyzed 3-*O*-α-L-rhamnoside of icariside II into icaritin (Fig. 4A, column B; Fig. 4C). However, the enzyme only hydrolyzed 7-*O*-β-D-glucoside of epimedim A or epimedin C into sagittatoside A or sagittatoside C, but did not hydrolyze the terminal 3-*O*-β-D-glucoside of sagittatoside A, or terminal 3-*O*-rhamnoside of sagittatoside C (Fig. 4A, column A and C). Thus, the hydrolysis pathways of the purified *Epimedium* flavonoid-glycosidase from *Aspergillus* sp.y848 strain on the glycosides of icariin, epimedin C, B and A are as shown in Fig. 5. The enzyme from *Aspergillus* sp.y848 strain is a special Epmedium flavonoid-glycosidase. The enzyme differs from the icariin-glycosidase from *Absidia* sp.E9r [19], and also from the previously reported crude snailase hydrolysis on *Epimedium* flavonoid-glycosides [20], β-glycosidase [22], thermophilic α-L-rhamnosidase and thermostable β-glucosidase [23].

Therefore, using the *Epimedium* flavonoid-glycosidase from *Aspergillus* sp.y848 strain, it is possible to prepare icaritin from icariin, which has a content ratio of over 58% of total flavonoids of *Epimedium* herb [5].

### Icaritin Preparation from Icariin by Crude Enzyme

As described above, the use of pure *Epimedium* flavonoid-glycosidase in the production of icaritin from icariin is costly. The pure enzyme yield was only 5.3% (U/U), and over 94.7% (U/U) enzyme was lost in the enzyme purification. Therefore, the low-cost crude enzyme from *Aspergillus* sp.y848 strain was used to prepare icaritin from icariin.

**Fig. 6:**
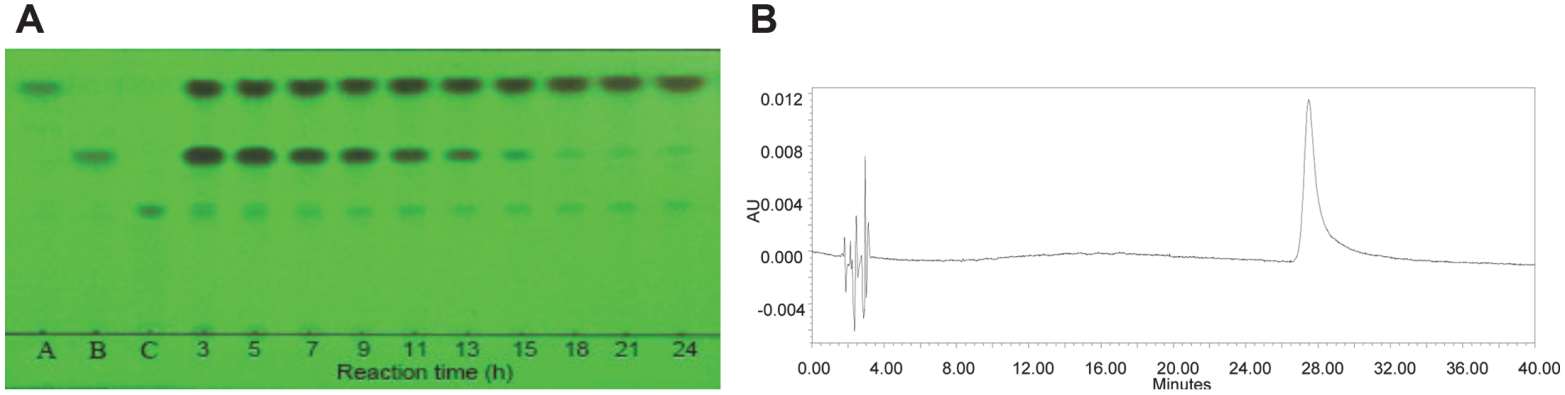
Icariin enzymatic conversion in different time in TLC and the icaritin product purity in HPLC. (**A**) Effects of different reaction time on 2.5% (w/v) icariin conversion at 40°C and pH 5.0 by crude enzyme in TLC. A, icaritin; B, icariside II; C, icariin; 3, 5,…to 24, reaction time (h). Developing solvent, ethyl acetate: butanone: methanol: water = 8: 7: 1: 1 (v/ v/v/v); rendering color in 280 nm ultraviolet. (**B**) Purity of icaritin product (0.04 mg/ml) in HPLC.

The crude enzyme optimum temperature was 40°C, and optimum pH was 5.0, which was the same as purified enzyme. The optimal substrate concentration of the enzyme reaction was examined. When 2.0, 2.5, 3.0, and 3.5%(w/v) icariin was reacted at 40°C using the crude enzyme, respectively; over 90% of 2.0% and 2.5% (w/v) into icaritin requires 18 h reaction time (Fig. 6A). In addition, over 90% of 3.0% (w/v) into icaritin needs 26 h reaction time, and over of 90% of 3.5% (w/v) icariin into icaritin needs 36 h. Therefore, the optimal reaction conditions for icaritin preparation from icariin by crude enzyme were identified as 2.5% icariin reaction at 40°C and pH 5.0 for 18 h.

Here, 10 g of icariin was dissolved in 200 ml of 0.02 M and pH 5.0 acetate buffer, mixed with 200 ml of crude enzyme. The mixture was then reacted at 40°C for 18-20 h, and over 95% (w/w) substrate was converted to icaritin, and the icaritin product was precipitated. The precipitated icaritin in the enzyme reaction solution was collected by freeze-centrifugation, washed three times with water, and dried to obtain dry crude icaritin. The dry crude icaritin was dissolved in tetrahydrofuran, filtered to remove un-dissolved material, and then a 3-4× volume of 50%methanol (v/v) was gradually added to precipitate icaritin, which was stored over night at room temperature. Then, the precipitated icaritin was collected by freeze-centrifugation, washed three times with cold 50% (v/v) methanol, and dried to get pure icaritin.

Furthermore, 5.04 g (13.68 mmol) pure icaritin was obtained from 10 g (14.78 mmol) icariin while the icaritin molar yield was 92.5%, and the weight yield was 50.4%. The above results are the average data from three experiments.

The purity of the icaritin product was examined by HPLC method according to HPLC method of The Pharmacopoeia of the Peoplés Republic of China [32]. The icaritin from enzymatic reaction and standard icaritin were dissolved in methanol to 0.04 mg/ml concentration. The flow rate was also 1 ml/min and the injected volume was 10 μl. There was one peak (retention time about 58 min) in the mobile phase of the *Epimedium*-flavonoid substrates and products in the enzyme reaction (the HPLC was omitted), and one peak (retention time about 28 min) in the mobile phase of methanol: tetrahydrofuran: 0.1% phosphoric acid water = 35: 26: 39 (Fig. 6B). In Fig. 6B showing icaritin HPLC, the theoretical plate number was about 328604, and the trailing factor was 1.99 [32]. The results of purified icaritin were the same as that of standard icaritin. Therefore, the purity of purified icaritin from enzyme reaction should be over 98% by HPLC.

Therefore, icaritin was successfully prepared for the first time from icariin by a non-GMO crude *Epimedium* flavonoid-glycosidase from the *Aspergillus* sp.y848 at low cost. The icaritin product is safe and can be used directly in health food, drugs and cosmetics. Moreover, our method is better than the previously reported methods of enzymatic icaritin preparation, such as that of icaritin preparation from icariin using icariin glycosidase from *Absidia* sp.E9r [19], or that of icaritin preparation from icariin using homemade β-glycosidase [22], and finally, that of icaritin preparation from epimedin C using transgenic thermophilic α-L-rhamnosidas and transgenic thermostable β-glucosidase [23]. Also, our result of 44.6% weight yield of icaritin was better than 4% weight yield of the thermostable enzyme from *Dictyoglomus thermophilum* DSM3960 [24].

### Structure of Icaritin Product

**Fig. 7:**
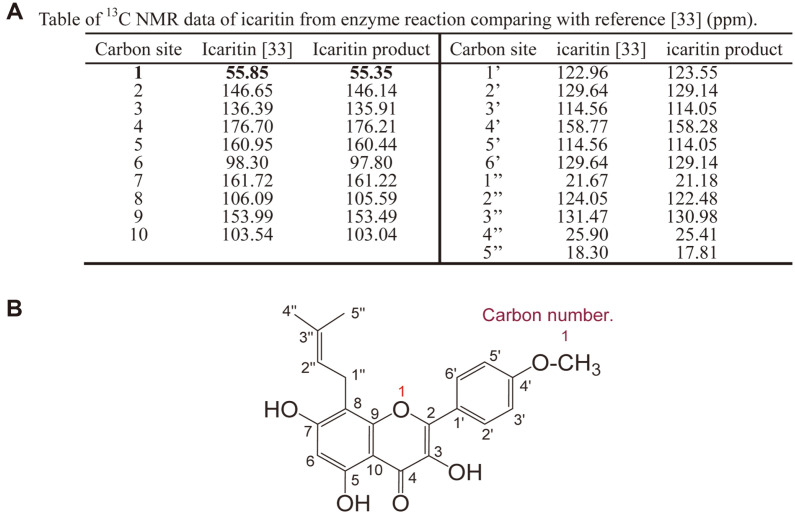
Table of ^13^C NMR data and structure of icaritin from enzyme reaction. (**A**) Table of ^13^C NMR data of icaritin from enzyme reaction comparing with reference [33] (ppm). (**B**) Structure of icaritin from enzyme reaction.

The NMR method was used to analyze the structure of icaritin from icariin by enzyme. The ^13^C NMR (150 MHz) spectral data for the icaritin (in Pyridine-*d_5_*) are shown in Fig. 7A and Fig. S5. In this work, assignments were based on 1H, 13C, DEPT, HMBC, HSQC, and COSY NMR experiments (Fig. S5) and compared with reference [33]. Based on previously reported NMR data on icaritin, the enzyme reaction product should be icaritin and the scientific name is 3,5,7-trihydroxy-2-(4-methoxyphenyl)- 8-(3-methyl-2-butenyl)- 4H-1- benzopyran-4-one (Fig. 7B).

## Discussion

A special *Epimedium* flavonoid-glycosidase that hydrolyzes multi-glycosides of icariin, epimedin C, B, and A was produced from the culture of *Aspergillus* sp.y848 strain. Optimal enzyme production was gained in a medium containing 5% (w/v) wheat bran extract and 0.7% (w/v) *Epimedium* leaf powder, and culture at 30°C for 6-7 days by 60-70 rpm stirring.

The enzyme was almost purified by a DEAE-cellulose column to pure enzyme, and showed a single peak in protein HPLC and a single spot in SDS-PAGE and PAGE. The enzyme’s molecular weight was approximately 73.2 kDa by SDS-PAGE. The optimal pH of the purified enzyme was 5.0 and the optimal temperature was 40°C. The *Km* and *Vmax* kinetic parameters of the enzyme were 15.63 mM and 55.56 mM/h for icariin. The purified enzyme hydrolyzed 7-*O*-β-D-glucoside of icariin to icariside II, and finally hydrolyzed 3-*O*-α-L-rhamonoside of icariside II into icaritin (aglycone). The enzyme also hydrolyzed 7-*O*-β-D-glucoside of epimedin B to sagittatoside B, and then further hydrolyzed terminal 3-*O*-β-D-xyloside of sagittatoside B to icariside II, and finally hydrolyzed 3-*O*-α-L-rhamonoside of icariside II into icaritin. The enzyme hydrolyzed 7-*O*-β-D-glucoside of epimedin C and A to sagittatoside A and C, but did not hydrolyze the terminal 3-*O*-α-L-rhamonoside of sagittatoside C or terminal 3-*O*-β-D-glucoside of sagittatoside A. Therefore, the enzyme from *Aspergillus* sp.y848 strain is a special *Epimedium* flavonoid-glycosidase. Using the enzyme, it is possible to prepare the icaritin with high pharmacological activities from the high-content icariin in *Epimedium* herb.

Given that the pure enzyme yield was only 5.3% (U/U), the crude *Epimedium* flavonoid-glycosidase from *Aspergillus* sp.y848 strain was used for icaritin preparation from icariin at low cost. When 2.5% (w/v) icariin was reacted at pH 5.0 and 40°C for 18-20 h with the crude enzyme in the optimal condition, 5.04 g (13.68 mmol) pure icaritin was obtained from 10 g (14.78 mmol) icariin and the icaritin molar yield was 92.5%, while the weight yield was 50.4%. The icaritin purity was 98% by HPLC.

In conclusion, icaritin was successfully produced via relatively simple and cost-effective methods from icariin using a non-GMO and low-cost crude enzyme prepared from the *Aspergillus* sp.y848 strain and such results have not been previously reported. other reports. Therefore, our results are meaningful for the development of medicines from *Epimedium* herb.

## Supplemental Materials

Supplementary data for this paper are available on-line only at http://jmb.or.kr.
