# Supplementary material for: Icaritin Preparation from Icariin by a Special Epimedium Flavonoid-Glycosidase from Aspergillus sp.y848 Strain
Source: J Microbiol Biotechnol. 2022 Feb 11;32(4):437–46. doi: 10.4014/jmb.2112.12036 (PMC9628809; doi:10.4014/jmb.2112.12036)
Supplement: Supplementary file 1 [file jmb-32-4-437-supple.pdf]

Supplementary Files

Icaritin Preparation from Icariin by a Special *Epimedium* Flavonoid-Glycosidase from *Aspergillus* sp.y848 Strain

* Corresponding author *E-mail addresses*: hongshan@dlpu.edu.cn (H. Yu), cksun110@vip.sina.com (C. Sun), fxjin@dlpu.edu.cn (F. Jin).

Fig. S1. Effect of enzyme inducer (*Epimedium* leaf powder) on enzyme production.

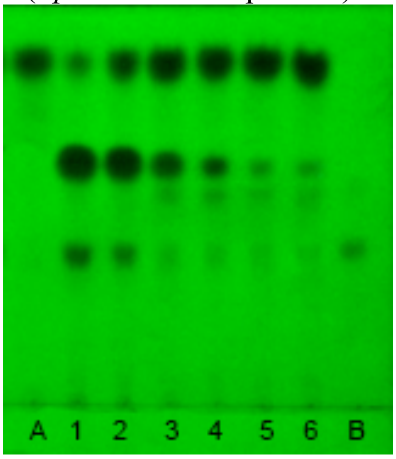

A, Icaririn; B, Icariin. 1, 0.2% *Epimedium* leaf powder； 2, 0.4% *Epimedium* leaf powder； 3, 0.5% *Epimedium* leaf powder； 4, 0.7% *Epimedium* leaf powder; 5, 0.7% *Epimedium* leaf powder； 6, 0.8% and 1% (w/v) *Epimedium* leaf powder. The 0.25% icariin was reacted by enzyme at 40 °C for 12 h. Developing solvent, ethyl acetate: butanone: methanol: water = 8: 7: 1: 1 (v/v/v/v); rendering colour in 280 nm ultraviolet.

The optimal enzyme inducer concentration was 0.7% *Epimedium* leaf powder.

Fig. S2. The fractions from DEAE-cellulose column in SDS-PAGE.

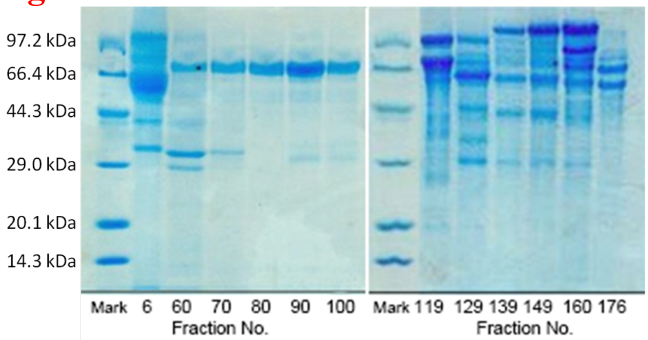

Mark, marker proteins including phosphatase b (97.4 kDa), bovine serum albumin (66.2 kDa), actin (43 kDa), carbonic anhydrase (31 kDa), trypsin inhibitor (22 kDa) and lysozyme (14.4 kDa).

Fig. S3. The enzyme molecular weight was calculated according to the mobility of marker proteins basing on plotting the log of the marker protein molecular weight [30] as showm in Fig. S2.

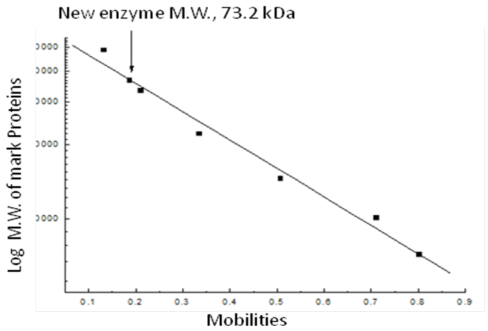

Fig. S4. Lineweaver–Burk plot of *Epimedium* flavonoid-glycosidase on the conversion of icariin. The *Km* and *Vmax* for icariin were 15.63 mM, and 55.56 mM/h; the enzyme reaction velocities (V_o) in 5 mM substrate were 13.5 mM/h..

The Michaelis constant (*Km*) and the maximal reaction velocity (*Vmax*) for *Epimedium* flavonoid-glycosidase from *Aspergillus* sp.y848 strain were measured with fully mixed icariin at concentrations of 12.5, 15.4, 20.0, 28.6, and 50.0 mM in 0.02 M acetate buffer (pH 5.0). The reaction was performed at 40 °C and pH 5.0 for 0.5, 1.0, 1.5, 2.0 and 3.0 h, respectively. The conversion velocities of icariin or epimedin A or epimedin B or epimedin C were obtained according to the TLC spot ratio of substrates and products in the enzyme reactions, respectively. The *Km* and *Vmax* were calculated by the Linweaver–Burk plots [32]. According to *Km* and *Vmax*, the conversion velocity *Vo* of icariin or epimedin A or epimedin B or epimedin C at a given substrate concentration was calculated using the Michaelis–Menten equation (1) [27-29]:

$$V = \frac{V_{max} \cdot [S]}{K_m + [S]} \qquad (1)$$

In the equation; [*S*] is the substrate concentration (mM) , *V* is the velocity (mM/h), *Km* is the Michaelis constant (mM), *Vmax* is the maximum velocity (mM/h). V_o (mM/h) is the conversion velocity at a given substrate concentration.

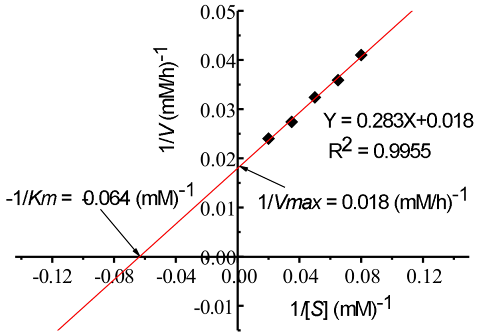

Table S1.
Effect of metallic ions on purified Epimedium flavonoid-glycosidase activity fo icariin (Relative activity %).

Concentration (mM/L)	Na^{+}	K^{+}	Mg^{2+}	Ca^{2+}	Zn^{2+}	Fe^{3+}	Cu^{2+}
0	100	100	100	100	100	100	100
5	99.5	99.6	110	106	109	20.1	60.1
10	105	104	116	108	90,1	5.05	45.0
50	98.5	99.8	114	105	60.0	0	10.1
100	107	101	109	103	49.0	0	0
200	102	102	106	102	5.0	0	0

Fig. S5. Additional information of spectra for enzymatic product icaritin analyzing by NMR

The structure of enzymatic product icaritin from icariin or *Epimedium*-flavonoids consisting of icariin, epimedin A and epimedin B and epimedin C was analyzed using NMR. The product was dissolved in Pyridine-d_5, and the NMR spectra were recorded by using the Bruke Avance 600 NMR spectrometer (^{1}H: 600 MHz; ^{13}C: 150 MHz) (Switzerland).

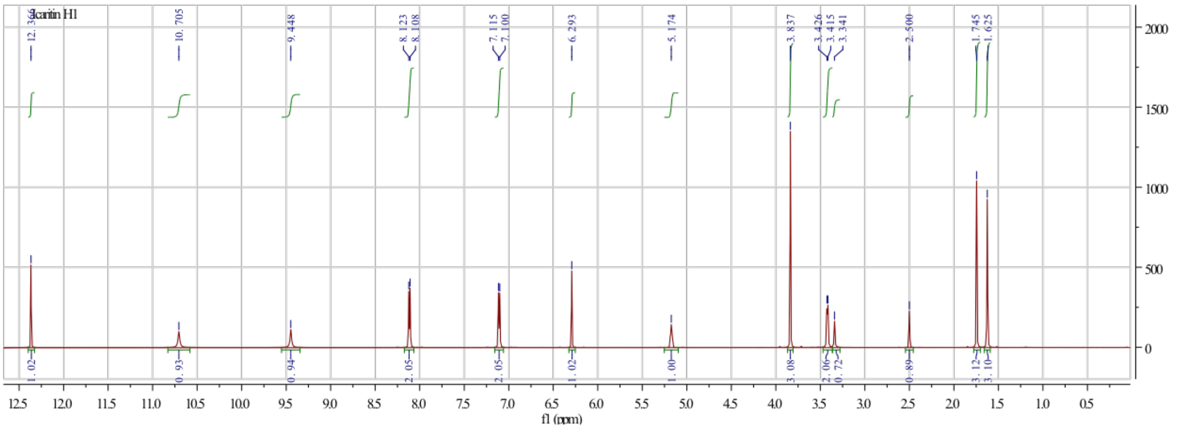

Fig. S5-1. ^{1}H NMR (600 MHz, Pyridine-D_5) spectrum of icaritin

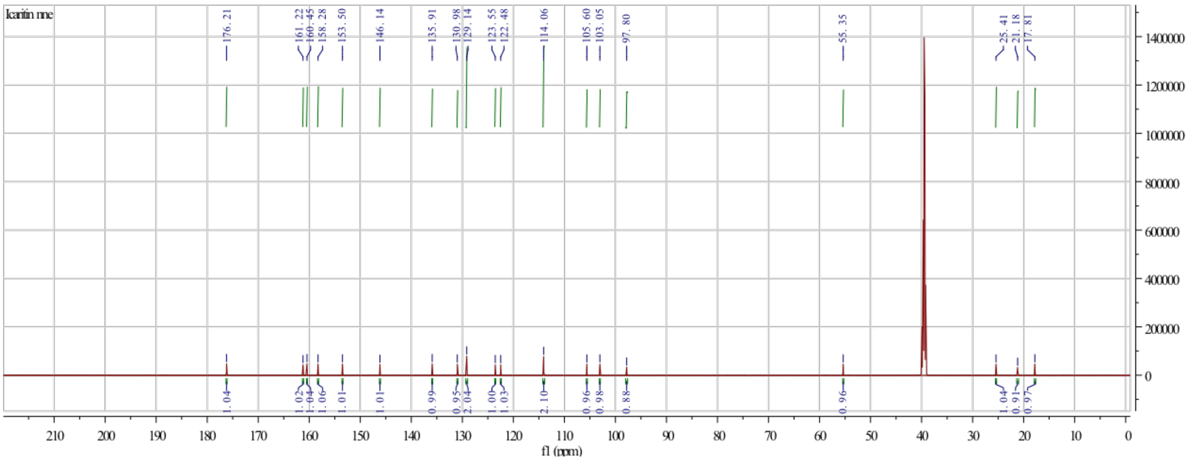

Fig. S5-2. ^{13}C NMR (150 MHz, Pyridine-D_5) spectrum of icaritin

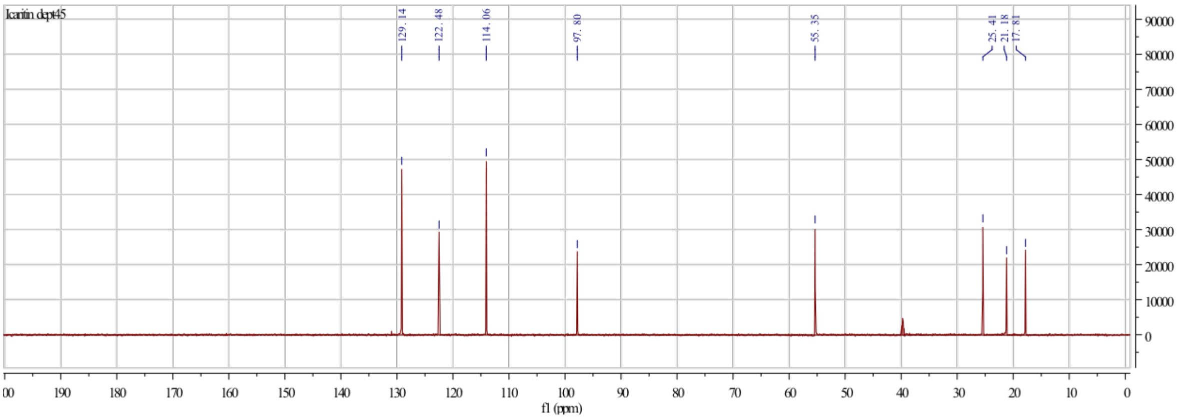

Fig. S5-3. ^{13}C DEPT 45 NMR spectrum of icaritin

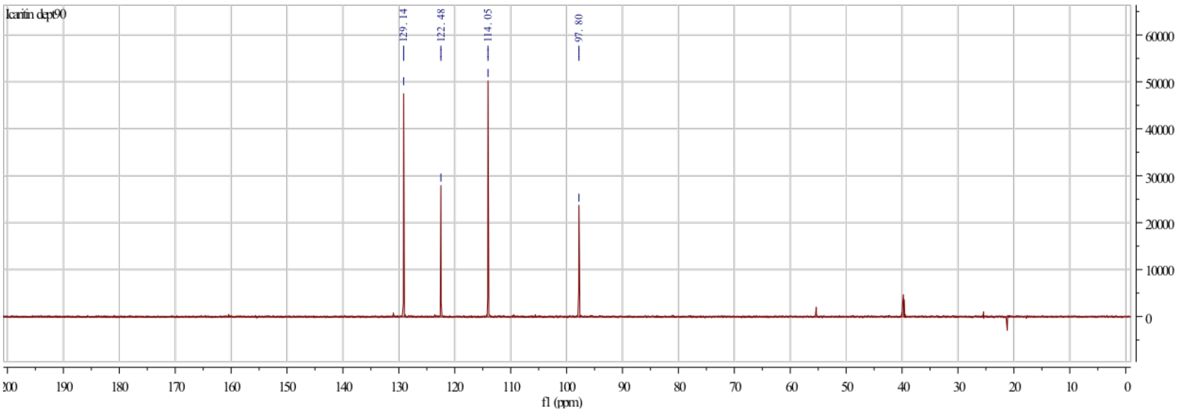

Fig. S5-4. ^{13}C DEPT 90 NMR spectrum of icaritin

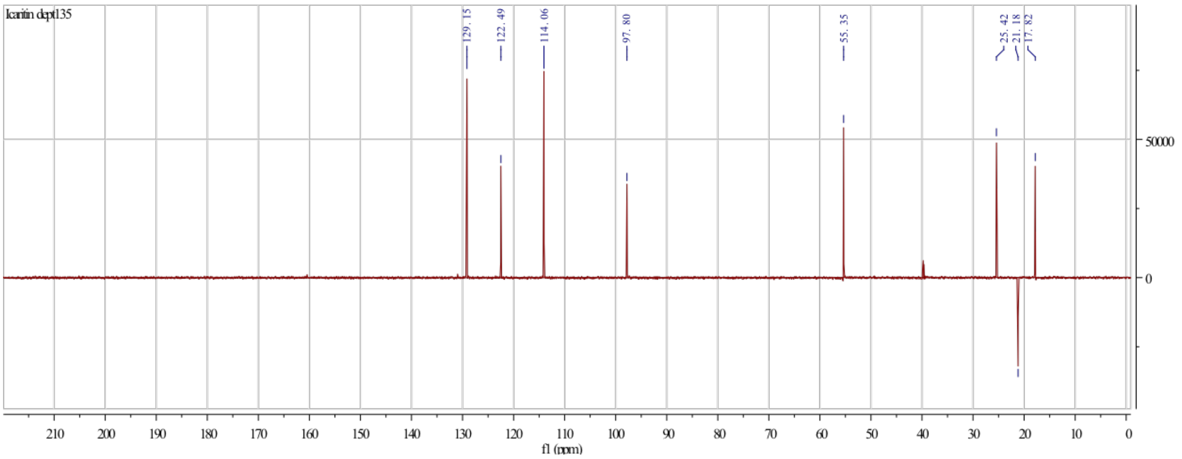

Fig. S5-5. ^{13}C DEPT 9135 NMR spectrum of icaritin

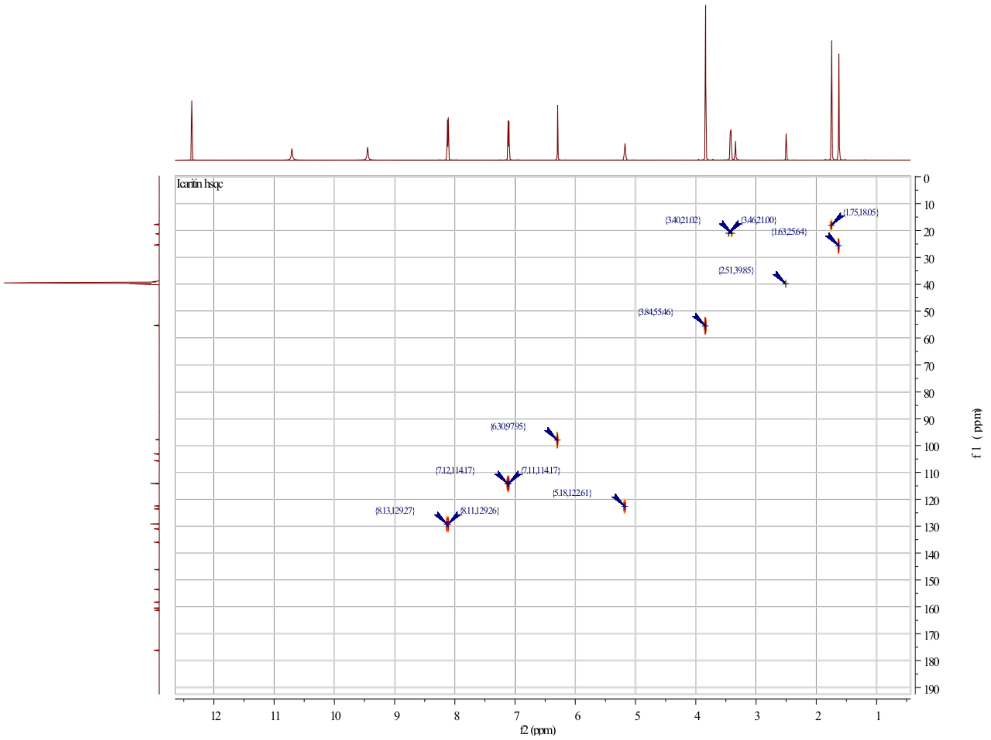

Fig. S5-6. ^{1}H-^{13}C HSQC spectrum of icaritin

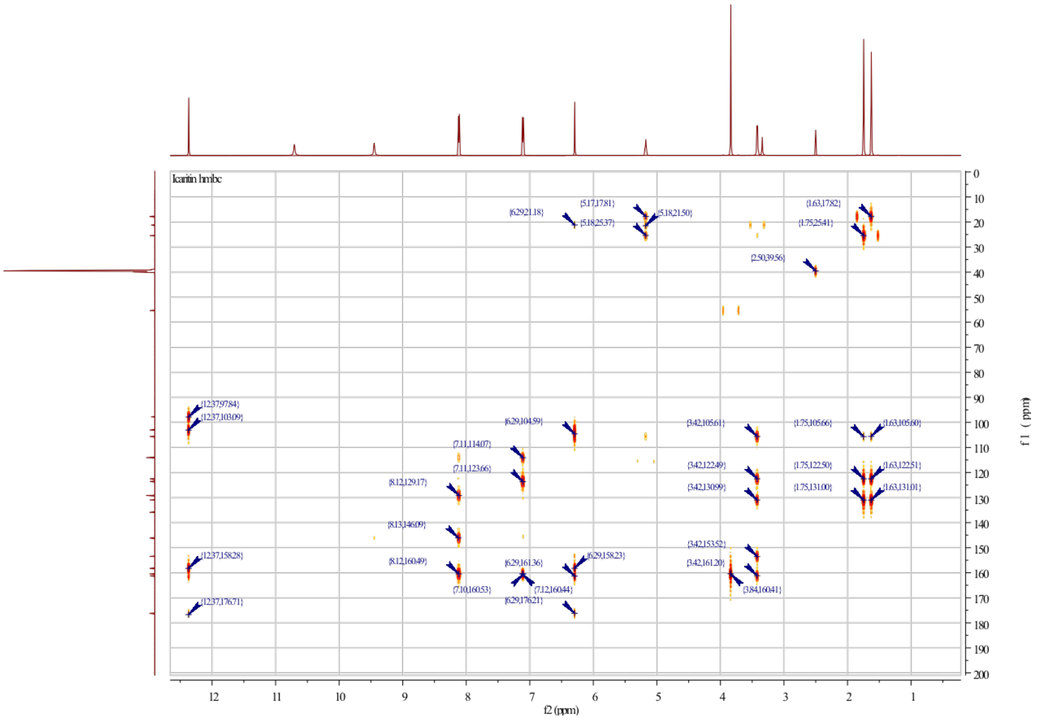

Fig. S5-7. ^{1}H-^{13}C HMBC spectrum of icaritin

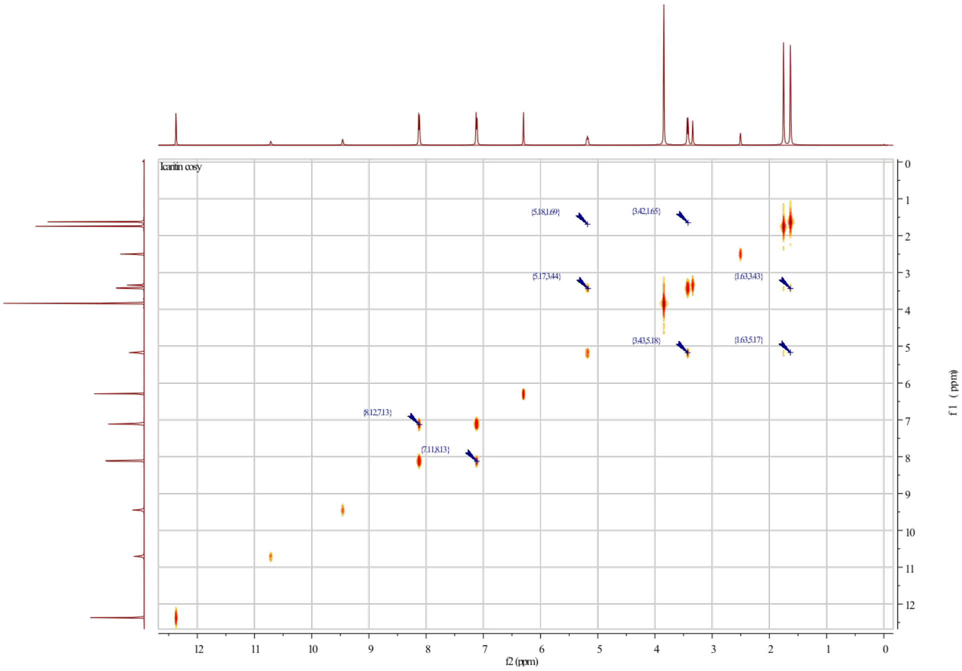

Fig. S5-8. ^{1}H-^{1}H COSY spectrum of icaritin
